# Robust Generalized Low Rank Approximations of Matrices

**DOI:** 10.1371/journal.pone.0138028

**Published:** 2015-09-14

**Authors:** Jiarong Shi, Wei Yang, Xiuyun Zheng

**Affiliations:** School of Science, Xi'an University of Architecture and Technology, Xi'an, China; University of East Piedmont, ITALY

## Abstract

In recent years, the intrinsic low rank structure of some datasets has been extensively exploited to reduce dimensionality, remove noise and complete the missing entries. As a well-known technique for dimensionality reduction and data compression, Generalized Low Rank Approximations of Matrices (GLRAM) claims its superiority on computation time and compression ratio over the SVD. However, GLRAM is very sensitive to sparse large noise or outliers and its robust version does not have been explored or solved yet. To address this problem, this paper proposes a robust method for GLRAM, named Robust GLRAM (RGLRAM). We first formulate RGLRAM as an *l*_1_-norm optimization problem which minimizes the *l*_1_-norm of the approximation errors. Secondly, we apply the technique of Augmented Lagrange Multipliers (ALM) to solve this *l*_1_-norm minimization problem and derive a corresponding iterative scheme. Then the weak convergence of the proposed algorithm is discussed under mild conditions. Next, we investigate a special case of RGLRAM and extend RGLRAM to a general tensor case. Finally, the extensive experiments on synthetic data show that it is possible for RGLRAM to exactly recover both the low rank and the sparse components while it may be difficult for previous state-of-the-art algorithms. We also discuss three issues on RGLRAM: the sensitivity to initialization, the generalization ability and the relationship between the running time and the size/number of matrices. Moreover, the experimental results on images of faces with large corruptions illustrate that RGLRAM obtains the best denoising and compression performance than other methods.

## Introduction

In the community of pattern recognition, machine learning and computer vision, a commonly-used tenet is that the interested datasets lie in single or multiple linear subspaces. This low rank structure can be exploited to reduce dimensionality, remove noise and complete the missing entries. In view of this, the low rank subspace methods have been attracting broad attentions in the past few years. These methods include not only the classical paradigms such as Principal Component Analysis (PCA) [1], Singular Value Decomposition (SVD) [2] and Linear Discriminant Analysis (LDA) [3], but also the recently emerged Low Rank Matrix Recovery (LRMR) composed mainly by Matrix Completion (MC) [4,5], Robust Principal Component Analysis (RPCA) [6,7] and Low Rank Representation (LRR) [8,9].

Conventionally, each sample is modeled by a vector and the collection of data samples is represented by a matrix. However, some real-world samples such as gray-level images are two-dimensional in essence. Under this circumstance, the traditional vector subspace model can not keep the bilinear structure of samples. To remedy this deficiency, one-dimensional subspace methods are successively generalized to the two-dimensional case. The resulting two-dimensional subspace methods mainly include two-dimensional PCA (2dPCA) [10], two-dimensional SVD (2dSVD) [11], two-dimensional LDA (2dLDA) [12], two-directional two-dimensional PCA ((2d)^2^PCA) [13], Generalized Low Rank Approximations of Matrices (GLRAM) [14,15] and so on. Among them, the latter two methods have the equivalent tri-factorization formulations, that is, they use two-sided transformations rather than single-sided ones. Compared to SVD, GLRAM is verified experimentally to have better compression performance and consume less computation time [14].

Recently, Liu et al. [15] revealed the relationship between GLRAM and SVD, and gave a lower-bound of GLRAM’s objective function; Shi et al. [16] proposed a method of matrix completion via GLRAM. The existing GLRAM is designed to handle Gaussian noise and the Frobenius norm is adopted to evaluate the magnitude of approximation errors. However, GLRAM does not work well in the presence of large sparse noise or outliers. As far as we know, the robustness of GLRAM does not have been explored or solved yet. To this end, this paper proposes a novel robust approach to GLRAM, named Robust GLRAM (RGLRAM). Mathematically, RGLRAM is formulated as an *l*_1_-norm minimization problem and the Augmented Lagrange Multiplier (ALM) method [17] is applied to solve this non-smooth optimization problem. We also extend RGLRAM to the tensor case and present an iterative scheme to robust low rank tensor approximations.

The rest of this paper is organized as follows. Section 2 briefly reviews GLRAM. Model and algorithm of RGLRAM are proposed respectively in Section 3 and 4. Section 5 presents the weak convergence results for the proposed algorithm under mild conditions. In Section 6, we discuss the special case of RGLRAM for a single matrix and generalize RGLRAM to the case of tensors. Experimental results on synthetic data and images of faces are reported in Section 7. Conclusions and directions for future work can be found in Section 8.

## Review of Generalized Low Rank Approximations of Matrices

As the generalization of SVD, Generalized Low Rank Approximations of Matrices (GLRAM) treats each sample as a two-dimensional matrix pattern and it simultaneously carries out tri-factorizations on multiple matrices. Given a collection of *N* two-dimensional training samples ${\left\{D_{i}\in R^{m\times n}\right\}}_{i=1}^{N}$, we decompose each sample into the product of three matrices:

$$D_{i}\approx LM_{i}R^{T},\;i=1,2,\ldots ,N\;,$$ (1)

where both $L\in R^{m\times r_{1}}$ and $R\in R^{n\times r_{2}}$ are orthogonal transformation matrices, $M_{i}\in R^{r_{1}\times r_{2}}$, max(*r*_1_, *r*_2_) < min(*m*, *n*). The goal of GLRAM is to seek *N*+2 matrices ***L***, ***R*** and ${\left\{M_{i}\right\}}_{i=1}^{N}$ such that ***LM***_*i*_ ***R*** ^*T*^ approximates ***D***_*i*_ for all *i*.

Mathematically, GLRAM can be formulated as a minimization problem:

$$\begin{array}{l} {\text{min}}_{L,{\left\{M_{i}\right\}}_{i=1}^{N},R}\;\sum_{i=1}^{N}{\left\|D_{i}-LM_{i}R^{T}\right\|}_{F}^{2}\\ \text{s}.\text{t}.\;L^{T}L=I_{r_{1}},R^{T}R=I_{r_{2}}, \end{array}$$ (2)

where $I_{r_{1}}$ is an *r*_1_×*r*_1_ identify matrix and ‖⋅‖_*F*_ is the Frobenius norm of a matrix. If {***L***, ***R***, ***M***_1_,…,***M***_*N*_} is the optimal solution of problem (2), then it holds that ***M***_*i*_ = ***L*** ^*T*^ ***D***_*i*_ ***R***. Based on this property, we only need to compute two transformations ***L*** and ***R*** by minimizing the reconstruction errors:

$$\begin{array}{l} {\text{min}}_{L,R}\;\sum_{i=1}^{N}{\left\|D_{i}-LL^{T}D_{i}RR^{T}\right\|}_{F}^{2}\\ \text{s}.\text{t}.\;L^{T}L=I_{r_{1}},R^{T}R=I_{r_{2}}. \end{array}$$ (3)

Generally speaking, the above optimization problem does not have a closed-form solution. An iterative procedure for computing ***L*** and ***R*** was proposed in [14]. Specifically, for fixed ***L***, the optimal ***R*** is computed by stacking the top *r*_2_ singular vectors of $\sum_{i=1}^{N}D_{i}^{T}LL^{T}D_{i}$. Similarly, for given ***R***, the optimal ***L*** is constructed by the top *r*_1_ singular vectors of $\sum_{i=1}^{N}D_{i}RR^{T}D_{i}^{T}$. The above iterative procedure is repeated until convergence. In this paper, the stopping condition is set as

$$\left(\sqrt{\sum_{i=1}^{N}{\left\|D_{i}-L^{\left(k\right)}M_{i}^{\left(k\right)}{\left(R^{\left(k\right)}\right)}^{T}\right\|}_{F}^{2}}-\sqrt{\sum_{i=1}^{N}{\left\|D_{i}-L^{\left(k+1\right)}M_{i}^{\left(k+1\right)}{\left(R^{\left(k+1\right)}\right)}^{T}\right\|}_{F}^{2}}\right)/\sqrt{\sum_{i=1}^{N}{\left\|D_{i}\right\|}_{F}^{2}}\leq \varepsilon ,$$ (4)

where *ε* is a small positive number.

GLRAM has good compression performance due to the fact that ***D***_*i*_ is approximated by ***LM***_*i*_ ***R*** ^*T*^. To store {***L***, ***R***, ***M***_1_,…,***M***_*N*_}, *mr*_1_ + *nr*_2_ + *Nr*_1_ *r*_2_ scalars are required. Hence, the compression ratio via GLRAM is *Nmn*/(*mr*_1_ + *nr*_2_ + *Nr*_1_ *r*_2_). Moreover, formulation (1) is equivalent to:

$$vec(D_{i})\approx (R⊗L)vec(M_{i}),\;i=1,2,\ldots ,N,$$ (5)

where ⊗ denotes the Kronecker product and *vec*(⋅) is a vectorization operator which stacks the columns of a matrix into a long column vector. Based on formulation (5), GLRAM can be interpreted as a linear representation problem, where ***R*** ⊗ ***L*** is the basis matrix.

## Robust Model of Generalized Low Rank Approximations of Matrices

We rewrite the tri-factorization formulation (1) as

$$D_{i}=LM_{i}R^{T}+E_{i},\;i=1,2,\ldots ,N,$$ (6)

where $E_{i}\in R^{m\times n}$ are noise matrices. If the entries of ***E***_*i*_ obey independent normal distributions with zero means, then their maximum likelihood estimator is equivalent to minimizing the objective function of problem (2). As is known to all, this objective function, also called cost function, is very sensitive to large sparse corruptions or outliers. For this purpose, we now assume that the entries of ***E***_*i*_ are generated by independent Laplacian distributions. According to the method of maximum likelihood estimation, the hidden factors {***L***, ***R***, ***M***_1_,…,***M***_*N*_} can be obtained by solving the following *l*_1_-norm minimization problem:

$$\begin{array}{l} {\text{min}}_{L,{\left\{M_{i}\right\}}_{i=1}^{N},R}\;\sum_{i=1}^{N}{\left\|D_{i}-LM_{i}R^{T}\right\|}_{1}\\ \text{s}.\text{t}.\;L^{T}L=I_{r_{1}},R^{T}R=I_{r_{2}}, \end{array}$$ (7)

where ‖⋅‖_1_ is the component-wise *l*_1_-norm of a matrix (i.e. the sum of absolute values of a matrix). To simplify the representation of the objective function in problem (7), we introduce *N* additional variables ***E***_1_,…, ***E***_*N*_. At this moment, the above minimization problem is equivalent to:

$$\begin{array}{l} {\text{min}}_{L,{\left\{M_{i}\right\}}_{i=1}^{N},R,{\left\{E_{i}\right\}}_{i=1}^{N}}\;\sum_{i=1}^{N}{\left\|E_{i}\right\|}_{1}\\ \text{s}.\text{t}.\;D_{i}=LM_{i}R^{T}+E_{i},\;i=1,2,\ldots ,N,\\ \;L^{T}L=I_{r_{1}},R^{T}R=I_{r_{2}}. \end{array}$$ (8)

We call problem (7) or (8) as Robust Generalized Low Rank Approximations of Matrices (RGLRAM).

Compared with problem (2), problem (7) or (8) is more robust to large sparse noise or outliers on account of the fact that the component-wise *l*_1_-norm of a matrix is the tightest convex relaxation of the component-wise *l*_0_-norm (i.e. the number of non-zero elements). Although problem (8) belongs to the *l*_1_-norm minimization, it is non-convex. Therefore, we can not directly use the available methods of compressed sensing to solve it. In the next section, we will apply the Augmented Lagrange Multipliers (ALM) method to solving problem (8).

## Algorithm to Robust Generalized Low Rank Approximations of Matrices

The method of Lagrange multipliers is a strategy for converting the optimization problem with equality constraints into an unconstrained problem. In view of this, we propose an iterative algorithm based on ALM to solve problem (8).

For the sake of simplicity, we set $M={\left\{M_{i}\right\}}_{i=1}^{N},E={\left\{E_{i}\right\}}_{i=1}^{N}$.Without considering the orthogonal constraints in problem (8), we construct its partial augmented Lagrange function:

$$\begin{array}{l} f_{\mu }(L,R,M,E,Y)\\ =\;\sum_{i=1}^{N}\left({\left\|E_{i}\right\|}_{1}+\langle Y_{i},D_{i}-LM_{i}R^{T}-E_{i}\rangle +\mu {\left\|D_{i}-LM_{i}R^{T}-E_{i}\right\|}_{F}^{2}/2\right)\\ =\sum_{i=1}^{N}\left({\left\|E_{i}\right\|}_{1}+\mu {\left\|D_{i}-LM_{i}R^{T}-E_{i}+Y_{i}/\mu \right\|}_{F}^{2}/2-{\left\|Y_{i}\right\|}_{F}^{2}/\left(2\mu \right)\right), \end{array}$$ (9)

where 〈⋅,⋅〉 indicates the inner product between matrices, the penalty factor *μ* is positive, $Y_{i}\in R^{m\times n}$ are Lagrange multiplier matrices and $Y={\left\{Y_{i}\right\}}_{i=1}^{N}$. For given ***Y***, a block-based gradient descent search technique is proposed to minimize *f*_*μ*_(***L***, ***R***, ***M***, ***E***, ***Y***). Concretely speaking, we let one block of variables be unknown and other blocks of variables be fixed at each iteration, and then the unknown block of variables is updated by minimizing *f*_*μ*_(***L***, ***R***, ***M***, ***E***, ***Y***). The specific update formulations are derived as follows.

**Computing *L*.** If ***L*** is unknown and other variables are fixed, we update ***L*** by minimizing *f*_*μ*_(***L***, ***R***, ***M***, ***E***, ***Y***) with respect to ***L***. Considering the orthogonal constraints in problem (8), we have

$$\begin{array}{l} {\left\|D_{i}-LM_{i}R^{T}-E_{i}+Y_{i}/\mu \right\|}_{F}^{2}\\ =\langle LM_{i}R^{T},LM_{i}R^{T}\rangle -2\langle LM_{i}R^{T},D_{i}-E_{i}+Y_{i}/\mu \rangle +{\left\|D_{i}-E_{i}+Y_{i}/\mu \right\|}_{F}^{2}\\ =-2\langle L,(D_{i}-E_{i}+Y_{i}/\mu )RM_{i}^{T}\rangle +{\left\|M_{i}\right\|}_{F}^{2}+{\left\|D_{i}-E_{i}+Y_{i}/\mu \right\|}_{F}^{2}. \end{array}$$ (10)

Hence, it holds that

$$\begin{array}{l} \;{\text{arg min}}_{L^{T}L=I_{r_{1}}}f_{\mu }(L,R,M,E,Y)\\ ={\text{arg min}}_{L^{T}L=I_{r_{1}}}{\sum_{i=1}^{N}\left\|D_{i}-LM_{i}R^{T}-E_{i}+Y_{i}/\mu \right\|}_{F}^{2}\\ ={\text{arg max}}_{L^{T}L=I_{r_{1}}}\langle L,P\rangle , \end{array}$$ (11)

where $P=\sum_{i=1}^{N}(D_{i}-E_{i}+Y_{i}/\mu )RM_{i}^{T}$.

The constraint surface $\left\{L\in R^{m\times r_{1}}|L^{T}L=I_{r_{1}}\right\}$ in problem (11) is known as the Stiefel manifold. For the optimization problems on Stiefel manifolds, Edelman et al. developed new Newton and conjugate gradient algorithms [18]. These two algorithms have heavy computation burden because they require the Hessian of the objective function. To reduce the computation complexity, we relax the orthogonal constraint into a sphere constraint and thus have

$${\text{max}}_{L^{T}L=I_{r_{1}}}\langle L,P\rangle \leq {\text{max}}_{{\left\|L\right\|}_{F}^{2}=r_{1}}\langle L,P\rangle .$$ (12)

It is obvious that the optimal solution of ${\text{max}}_{{\left\|L\right\|}_{F}^{2}=r_{1}}\langle L,P\rangle$ is $\sqrt{r_{1}}P/{\left\|P\right\|}_{F}$. Meanwhile, we can verify that $L=\sqrt{r_{1}}P/{\left\|P\right\|}_{F}$ does not increase the value of *f*_*μ*_(***L***, ***R***, ***M***, ***E***, ***Y***) although it probably does not satisfy the orthogonal constraint. Then we set

$$L:=QR(\sqrt{r_{1}}P/{\left\|P\right\|}_{F})=QR(P),$$ (13)

where *QR*(⋅) means the thin QR decomposition on a matrix and ***L*** is an orthonormal basis of the range space of ***P***. It can not be guaranteed that the iteration formulation (13) decreases the value of *f*_*μ*_(***L***, ***R***, ***M***, ***E***, ***Y***). Since

$$\left(\sqrt{r_{1}}P/{\left\|P\right\|}_{F}\right)M_{i}R^{T}=\left(\sqrt{r_{1}}LL^{T}P/{\left\|P\right\|}_{F}\right)M_{i}R^{T}=L\left(\sqrt{r_{1}}L^{T}P/{\left\|P\right\|}_{F}\right)M_{i}R^{T},$$ (14)

the value of *f*_*μ*_(***L***, ***R***, ***M***, ***E***, ***Y***) surely does not increases after updating ***M***_*i*_.

**Computing *M*.** When ***M***_*j*_ is unknown and other blocks of variables are given, the update formulation for ***M***_*j*_ is calculated as:

$$\begin{array}{l} M_{j}:={\text{arg min}}_{M_{j}}f_{\mu }(L,R,M,E,Y)\\ ={\text{arg min}}_{M_{i}}{\left\|LM_{j}R^{T}-\left(D_{j}-E_{j}+Y_{j}/\mu \right)\right\|}_{F}^{2}\\ ={\text{arg min}}_{M_{j}}\langle LM_{j}R^{T},LM_{j}R^{T}\rangle -2\langle LM_{j}R^{T},D_{j}-E_{j}+Y_{j}/\mu \rangle \\ ={\text{arg min}}_{M_{j}}\langle M_{j},M_{j}\rangle -2\langle M_{j},L^{T}\left(D_{j}-E_{j}+Y_{j}/\mu \right)R\rangle \\ =L^{T}\left(D_{j}-E_{j}+Y_{j}/\mu \right)R. \end{array}$$ (15)

**Computing *R*.** For each *j* ∈ {1, 2,…, *N*}, we have

$${\left\|D_{j}-LM_{j}R^{T}-E_{j}+Y_{j}/\mu \right\|}_{F}^{2}={\left\|{\left(D_{j}-E_{j}+Y_{j}/\mu \right)}^{T}-RM_{j}^{T}L^{T}\right\|}_{F}^{2}.$$ (16)

This equation means that the iterative formulation of ***R*** is similar with that of ***L***. Let $T=\sum_{i=1}^{N}{\left(D_{i}-E_{i}+Y_{i}/\mu \right)}^{T}LM_{i}$. Then ***R*** is updated as

R:=QR(T). (17)

It is worth noting that Eq (17) is followed by the calculation procedure of ***M*** in order to decrease the value of *f*_*μ*_(***L***, ***R***, ***M***, ***E***, ***Y***).

**Computing *E*.** Fix ***L***, ***R***, ***M*** and ***Y***, and minimize *f*_*μ*_(***L***, ***R***, ***M***, ***E***, ***Y***) with respect to ***E***_*j*_:

$$\begin{array}{l} E_{j}:={\text{arg min}}_{E_{j}}\;f_{\mu }(L,R,M,E,Y)\\ ={\text{arg min}}_{E_{j}}\;{\left\|E_{j}\right\|}_{1}/\mu +{\left\|D_{j}-LM_{j}R^{T}-E_{j}+Y_{j}/\mu \right\|}_{F}^{2}/2\\ =S_{1/\mu }\left(D_{j}-LM_{j}R^{T}+Y_{j}/\mu \right), \end{array}$$ (18)

where $S_{\delta }\left(\cdot \right):R^{m\times n}\to R^{m\times n}$ is an absolute value shrinkage operator defined by

$${\left(S_{\delta }\left(X\right)\right)}_{ij}=\left\{ \begin{array}{l} \left|x_{ij}\right|-\delta ,\;\text{if}\;\left|x_{ij}\right|\geq \delta ,\\ 0,\;\text{otherwise}, \end{array}\right.$$ (19)

for arbitrary real matrix ***X*** = (*x*_*ij*_)_*m*×*n*_ and *δ* > 0.

**Computing *Y*.** For given ***L***, ***R***, ***M*** and ***E***, we calculate ***Y***_*j*_ according to the following formulation

$$Y_{j}:=Y_{j}+\mu \left(D_{j}-LM_{j}R^{T}-E_{j}\right).$$ (20)

Denote the *m*×*n* zero matrix by ***O***_*m*×*n*_. The whole iterative procedure is outlined as follows.

Algorithm: ALM algorithm for RGLRAM

Input: Data matrices ${\left\{D_{i}\right\}}_{i=1}^{N}$, *r*_1_ and *r*_2_.

Initialize: ***L***, ***R***, ***M***_*i*_ = ***L*** ^*T*^ ***D***_*i*_ ***R***, ***E***_*i*_ = ***O***_*m*×*n*_, ***Y***_*i*_ = ***O***_*m*×*n*_, *ρ* = 1.1, *μ* = 10^−4^, *μ*_max_ = 10^10^, *i* = 1, 2,…, *N*.

Step 1: while not converged do

Update ***L*** according to (13);

Update ***M*** according to (15);

Update ***R*** according to (17);

Update ***M*** according to (15);

Update ***E*** according to (18);

Update ***Y*** according to (20);

Update *μ* as *μ*:= min(*ρμ*, *μ*_max_);

End while.

Output: $L,R,{\left\{M_{i}\right\}}_{i=1}^{N}$ and ${\left\{E_{i}\right\}}_{i=1}^{N}$.

During the initialization, we can stipulate ***L*** = orth(randn(*m*,*r*_1_)) and ***R*** = orth(randn(*n*,*r*_2_)), where orth(⋅) is an orthonormal basis operator for the range of a matrix and randn(*m*, *r*_1_) is an *m*×*r*_1_ random matrix generated by the standard normal distribution. The stopping condition of can be set as

$$\sqrt{\sum_{i=1}^{N}{\left\|D_{i}-LM_{i}R^{T}-E_{i}\right\|}_{F}^{2}}/\sqrt{\sum_{i=1}^{N}{\left\|D_{i}\right\|}_{F}^{2}}\leq \varepsilon ,$$ (21)

or the maximum number of iterations is reached, where *ε* is a sufficiently small positive number.

Most of the time spent by the above algorithm is in the matrix multiplication and the thin QR decomposition. For the convenience of description, we suppose that *m* ≥ *n* > *r*_1_ ≥ *r*_2_. Under the assumption, the computation complexity of GLRAM at each loop is *O*(*Nmnr*_2_). Subsequently, we discuss the computation complexity of the ALM algorithm. It takes *O*(*Nmnr*_2_) time for computing ***P*** and $O(mr_{1}^{2})$ time for performing the *QR* decomposition on ***P***. Hence, the computation time of ***L*** is *O*(*Nmnr*_2_). Similarly, the computation time of ***R*** is also *O*(*Nmnr*_2_). Moreover, the computation times of ***M***, ***E*** and ***Y*** are all *O*(*Nmnr*_2_). In conclusion, the total computation complexity of the ALM algorithm at each loop is *O*(*Nmnr*_2_), which means that RGLRAM has the same computation complexity as RGLRAM.

## Convergence Analysis on the Proposed Algorithm

To the best of our knowledge, the convergence theory of ALM algorithms has not been established on non-convex problems or convex problems with more than two blocks of variables. Consequently, it is very difficult for us to prove the convergence of the proposed ALM algorithm. However, empirical evidence in Section 7 shows the strong convergence behavior of our algorithm. This section will discuss the weak convergence results of the proposed algorithm under mild conditions.

If both ***L*** and ***R*** are known, then problem (8) is reformulated as

$$\begin{array}{l} {\text{min}}_{{\left\{M_{i}\right\}}_{i=1}^{N},{\left\{E_{i}\right\}}_{i=1}^{N}}\;\sum_{i=1}^{N}{\left\|E_{i}\right\|}_{1}\\ \text{s}.\text{t}.\;D_{i}=LM_{i}R^{T}+E_{i},\;i=1,2,\ldots ,N. \end{array}$$ (22)

The augmented Lagrange function of the above problem is

$${\tilde{f}}_{\mu }(M,E,Y)=\;\sum_{i=1}^{N}\left({\left\|E_{i}\right\|}_{1}+\langle Y_{i},D_{i}-LM_{i}R^{T}-E_{i}\rangle +\mu {\left\|D_{i}-LM_{i}R^{T}-E_{i}\right\|}_{F}^{2}/2\right).$$ (23)

To solve problem (22), we can simply revise the ALM algorithm by deleting the iterative formulations of ***L*** and ***R***. In such a case, we have the following statement.

**Theorem 1.** Let {***M*** ^(*k*)^, ***E*** ^(*k*)^, ***Y*** ^(*k*)^} be the sequences produced by the revised ALM algorithm. If ${\tilde{f}}_{0}(M,E,Y)=\;\sum_{i=1}^{N}\left({\left\|E_{i}\right\|}_{1}+\langle Y_{i},D_{i}-LM_{i}R^{T}-E_{i}\rangle \right)$ has a saddle point, then the sequences {***M*** ^(*k*)^, ***E*** ^(*k*)^} satisfy that the objective function in problem (22) approaches the optimal value as *k* → ∞.

***Proof*.** The objective function of problem (22) can be rewritten as *g*(***M***) + *h*(***E***), where *g*(***M***) = 0 and $h(E)=\;\sum_{i=1}^{N}{\left\|E_{i}\right\|}_{1}$. It is obvious that *g*(***M***) and *h*(***E***) are two closed, proper and convex functions.

Let ***d*** = *vec*((***D***_1_,…, ***D***_*N*_)), ***x*** = *vec*((***M***_1_,…, ***M***_*N*_)), ***y*** = *vec*((***E***_1_,…, ***E***_*N*_)) and ***B*** = *diag*(***R***⊗***L***,…,***R***⊗***L***) $\in R^{mnN\times r_{1}r_{2}N}$, where *diag*(⋅) is the diagonal block matrix operator. Then the equality constraints in problem (22) can be re-expressed as ***Bx*** + ***y*** = ***d***. We denote *g*(***x***) = *g*(***M***) and *h*(***y***) = *h*(***E***). According to the basic convergence result given in [19], we have $\underset{k\to \infty }{\text{lim}}\left(D_{j}^{\left(k\right)}-LM_{j}^{\left(k\right)}R^{T}-E_{j}^{\left(k\right)}\right)=0$ and $\underset{k\to \infty }{\lim}\;g(M^{\left(k\right)})+h(E^{\left(k\right)})=f^{*}$, where *f* ^*^ is the minimization value of *g*(***M***) + *h*(***E***) under the constraints of ***Bx*** + ***y*** = ***d***. This ends the proof.

Moreover, problem (8) is essentially equivalent to the following minimization problem without orthogonal constraints:

$$\begin{array}{l} {\text{min}}_{L,M,R,E}\;\sum_{i=1}^{N}{\left\|E_{i}\right\|}_{1}\\ \text{s}.\text{t}.\;D_{i}=LM_{i}R^{T}+E_{i},\;i=1,2,\ldots ,N. \end{array}$$ (24)

Its un-augmented Lagrange function is

$$\varphi (L,R,M,E,Y)=\;\sum_{i=1}^{N}\left({\left\|E_{i}\right\|}_{1}+\langle Y_{i},D_{i}-LM_{i}R^{T}-E_{i}\rangle \right).$$ (25)

On the basis of the differentials or sub-differentials of *φ*(***L***, ***R***, ***M***, ***E***, ***Y***) with respect to each block of variables, we have the following KKT conditions for problem (24):

$$\left\{ \begin{array}{l} \sum_{i=1}^{N}Y_{i}RM_{i}^{T}=0,\\ \sum_{i=1}^{N}Y_{i}^{T}LM_{i}=0,\\ L^{T}Y_{j}R=0,\\ D_{j}=LM_{j}R^{T}+E_{j},\\ Y_{j}\in \partial {\left\|E_{j}\right\|}_{1},\;j=1,2,\ldots ,N, \end{array}\right.$$ (26)

where ∂‖⋅‖_1_ is the set of sub-differentials of the component-wise *l*_1_-norm of a matrix.

For arbitrary *μ* > 0, the condition ***Y***_*j*_ ∈ ∂‖***E***_*j*_‖_1_ is equivalent to

$$E_{j}+\frac{1}{\mu }Y_{j}\in E_{j}+\frac{1}{\mu }\partial {\left\|E_{j}\right\|}_{1}.$$ (27)

Denote $Q_{\mu }(t)=t+\frac{1}{\mu }\partial \left|t\right|$. Then *Q*_*μ*_(*t*) is monotonically increasing so that $Q_{\mu }^{-1}(t)=S_{1/\mu }(t)$ [20]. We apply element-wise *Q*_*μ*_(*t*) to ***E***_*j*_ and have $Q_{\mu }(E_{j})=E_{j}+\frac{1}{\mu }\partial {\left\|E_{j}\right\|}_{1}$. Hence, we arrive at

$$E_{j}=Q_{\mu }^{-1}\left(E_{j}+\frac{1}{\mu }Y_{j}\right)=S_{1/\mu }\left(E_{j}+\frac{1}{\mu }Y_{j}\right)=S_{1/\mu }\left(D_{j}-LM_{j}R^{T}+\frac{1}{\mu }Y_{j}\right)$$ (28)

according to the equation ***D***_*j*_ = ***LM***_*j*_ ***R*** ^*T*^ + ***E***_*j*_. The above analysis means that the condition ***Y***_*j*_ ∈ ∂‖***E***_*j*_‖_1_ in (26) can be replaced by (28).

**Theorem 2.** Let ***X*** = {***L***, ***R***, ***M***, ***E***, ***Y***} and ${\left\{X^{\left(k\right)}\right\}}_{k=1}^{\infty }$ be an iterative sequence produced by the ALM algorithm. If $\underset{k\to \infty }{\text{lim}}{\left\|X^{\left(k+1\right)}-X^{\left(k\right)}\right\|}_{F}=0$, $\underset{k\to \infty }{\text{lim}}\sum_{i=1}^{N}Y_{i}^{\left(k\right)}R^{\left(k\right)}{\left(M_{i}^{\left(k\right)}\right)}^{T}=0$ and $\underset{k\to \infty }{\text{lim}}\sum_{i=1}^{N}{\left(Y_{i}^{\left(k\right)}\right)}^{T}L^{\left(k\right)}M_{i}^{\left(k\right)}=0$, then any accumulation point of ${\left\{X^{\left(k\right)}\right\}}_{k=1}^{\infty }$ satisfies the KKT conditions (26), where

$${\left\|X\right\|}_{F}^{2}={\left\|L\right\|}_{F}^{2}+{\left\|R\right\|}_{F}^{2}+\sum_{i=1}^{N}\left({\left\|M_{i}\right\|}_{F}^{2}+{\left\|E_{i}\right\|}_{F}^{2}+{\left\|Y_{i}\right\|}_{F}^{2}\right),\;X^{\left(k\right)}=\left\{L^{\left(k\right)},R^{\left(k\right)},M^{\left(k\right)},E^{\left(k\right)},Y^{\left(k\right)}\right\}.$$

***Proof*.** By the iterative formulations (18) and (20), we have

$$E_{j}^{\left(k+1\right)}-E_{j}^{\left(k\right)}=S_{1/\mu }\left(D_{j}-L^{\left(k\right)}M_{j}^{\left(k\right)}{\left(R^{\left(k\right)}\right)}^{T}+\frac{1}{\mu }Y_{j}^{\left(k\right)}\right)-E_{j}^{\left(k\right)}\;,$$ (29)

$$Y_{j}^{\left(k+1\right)}-Y_{j}^{\left(k\right)}=\mu \left(D_{j}-L^{\left(k\right)}M_{j}^{\left(k\right)}{\left(R^{\left(k\right)}\right)}^{T}-E_{j}^{\left(k\right)}\right).$$ (30)

The term $\underset{k\to \infty }{\text{lim}}{\left\|X^{\left(k+1\right)}-X^{\left(k\right)}\right\|}_{F}=0$ implies that both sides of (29) and (30) tend to zeros as *k* approaches to infinity. Therefore, it holds that

$$\underset{k\to \infty }{\text{lim}}S_{1/\mu }\left(D_{j}-L^{\left(k\right)}M_{j}^{\left(k\right)}{\left(R^{\left(k\right)}\right)}^{T}+\frac{1}{\mu }Y_{j}^{\left(k\right)}\right)-E_{j}^{\left(k\right)}=0\;,$$ (31)

$$\underset{k\to \infty }{\text{lim}}D_{j}-L^{\left(k\right)}M_{j}^{\left(k\right)}{\left(R^{\left(k\right)}\right)}^{T}-E_{j}^{\left(k\right)}=0.$$ (32)

Moreover, by

$$\begin{array}{l} M_{j}^{\left(k+1\right)}-M_{j}^{\left(k\right)}={\left(L^{\left(k\right)}\right)}^{T}(D_{j}-E_{j}^{\left(k\right)}+Y_{j}^{\left(k\right)}/\mu )R^{\left(k\right)}-M_{j}^{\left(k\right)}\\ ={\left(L^{\left(k\right)}\right)}^{T}\left(D_{j}-E_{j}^{\left(k\right)}-L^{\left(k\right)}M_{j}^{\left(k\right)}{\left(R^{\left(k\right)}\right)}^{T}\right)R^{\left(k\right)}+{\left(L^{\left(k\right)}\right)}^{T}\left(L^{\left(k\right)}M_{j}^{\left(k\right)}{\left(R^{\left(k\right)}\right)}^{T}+Y_{j}^{\left(k\right)}/\mu \right)R^{\left(k\right)}-M_{j}^{\left(k\right)}\\ ={\left(L^{\left(k\right)}\right)}^{T}\left(D_{j}-E_{j}^{\left(k\right)}-L^{\left(k\right)}M_{j}^{\left(k\right)}{\left(R^{\left(k\right)}\right)}^{T}\right)R^{\left(k\right)}+{\left(L^{\left(k\right)}\right)}^{T}\left(Y_{j}^{\left(k\right)}/\mu \right)R^{\left(k\right)}\;, \end{array}$$ (33)

we have $\underset{k\to \infty }{\text{lim}}{\left(L^{\left(k\right)}\right)}^{T}\;Y_{j}^{\left(k\right)}R^{\left(k\right)}=0$. This completes the proof.

## Special Case and Tensor Extension of RGLRAM

This section will conduct a further discussion on RGLRAM from two aspects. Firstly, we consider the robust low rank approximations of a single matrix. Secondly, we study the robust low rank tensor approximations by generalizing the proposed method to higher order tensors.

### The Case for *N* = 1

Now, we consider a special case of RGLRAM, that is, *N* = 1. Under this case, problem (8) is rewritten as

$$\begin{array}{l} {\text{min}}_{L,M_{1},R}\;{\left\|D_{1}-LM_{1}R^{T}\right\|}_{1}\\ \text{s}.\text{t}.\;L^{T}L=I_{r_{1}},R^{T}R=I_{r_{2}}. \end{array}$$ (34)

We call this problem as *l*_1_-norm SVD. In other words, problem (34) is the robust version of SVD. Recently, Wright et al. [6] and Candès et al. [7] proposed a principal component pursuit method to recover simultaneously the low rank and the sparse components. The proposed method, also referred to as Robust PCA (RPCA), is described mathematically as a convex optimization problem:

$$\begin{array}{l} {\text{min}}_{A,E_{1}}\;{\left\|A\right\|}_{*}+\lambda {\left\|E_{1}\right\|}_{1}\\ \text{s}.\text{t}.\;D_{1}=A+E_{1}, \end{array}$$ (35)

where ‖***A***‖_*_ is the nuclear norm of ***A*** (i.e. the sum of singular values of ***A***) and the tradeoff parameter *λ* > 0. A good rule of thumb for determining the parameter *λ* is that $\lambda =1/\sqrt{\text{max}(m,n)}$ [7]. The main algorithms for solving the nuclear minimization problem (35) suffer from high computation burden of SVD at each iteration. If the rank of the low rank component is properly estimated, then RPCA is boiled down to problem (34).

We further set *r*_1_ = *r*_2_ = *r*. Then problem (34) is transformed into

$${\text{min}}_{U,V}\;{\left\|D_{1}-UV^{T}\right\|}_{1},$$ (36)

where $U\in R^{m\times r},V\in R^{n\times r}$. This problem is also named as *l*_1_-norm PCA. An alternative optimization method was proposed to solve it [21]. Specifically, we minimize one argument ***U*** or ***V*** while keeping the other one fixed and then the optimization problem is decomposed into *m* or *n* independent linear programmings. Kwak [22] proposed another method of PCA based on *l*_1_-norm maximization. This method changes problem (36) into the following formulation

$${\text{max}}_{U}\;{\left\|U^{T}D_{1}\right\|}_{1},\;\text{s}.\text{t}.\;U^{T}U=I_{r}.$$ (37)

Although ***V*** is absent in the above problem, it is still very hard to directly solve it. A greedy strategy [22] was proposed to solve problem (37). However, the successive greedy solutions might not be ideal.

### Extensions to Higher Order Tensors

A *K*-order tensor is defined as $X\in R^{I_{1}\times I_{2}\times \cdots \times I_{K}}$ whose (*i*_1_, *i*_2_,…, *i*_*K*_) entry is $x_{i_{1}i_{2}\ldots i_{K}}$, where 1 ≤ *i*_*k*_ ≤ *I*_*k*_, 1 ≤ *k* ≤ *K*. The *k*-mode matricization of X is defined as $X_{\left(k\right)}\in R^{I_{k}\times (I_{1}\cdots I_{k-1}I_{k+1}\cdots I_{K})}$. Given a matrix $U\in R^{J_{k}\times I_{k}}$, the *k*-mode product of X by ***U***, denoted as $X\times_{k}U$, is an *I*_1_×…×*I*_*k*−1_×*J*_*k*_×*I*_*k*+1_×…×*I*_*K*_ tensor whose (*i*_1_,…,*i*_*k*−1_,*j*,*i*_*k+*1_,…,*i*_*K*_) entry is computed by $\sum_{i_{k}=1}^{I_{k}}x_{i_{1}i_{2}\ldots i_{K}}u_{ji_{k}}$. The inner product, component-wise *l*_1_-norm and Frobenius norm of matrices can be easily generalized to tensors. Refer to the survey [23] for further understanding on tensor algebra.

In the following, we first consider the tensor expression of RGLRAM. Three-order tensors D, M and E are formed by concentrating the collections of ${\left\{D_{i}\right\}}_{i=1}^{N}$, ${\left\{M_{i}\right\}}_{i=1}^{N}$ and ${\left\{E_{i}\right\}}_{i=1}^{N}$ respectively, where $D(:,:,i)=D_{i},M(:,:,i)=M_{i},E(:,:,i)=E_{i},i=1,2,\ldots ,N$. Hence, Eq (6) can be re-expressed as $D=M\times_{1}L\times_{2}R+E$. Based on this, problem (8) is reformulated as

$$\begin{array}{l} {\text{min}}_{L,M,R,E}\;{\left\|E\right\|}_{1}\\ \text{s}.\text{t}.\;D=M\times_{1}L\times_{2}R+E,\\ \;L^{T}L=I_{r_{1}},R^{T}R=I_{r_{2}}. \end{array}$$ (38)

The corresponding partial augmented Lagrange function is

$$f_{\mu }(L,R,M,E,Y)={\left\|E\right\|}_{1}+\mu {\left\|D-M\times_{1}L\times_{2}R-E\right\|}_{F}^{2}/2+\langle Y,D-M\times_{1}L\times_{2}R-E\rangle$$ (39)

where $Y\in R^{m\times n\times N}$ is the Lagrange multiplier tensor.

Next, we extend RGLRAM to the general case of tensors and propose the corresponding iterative scheme to robust low rank tensor approximations. Consider a *K*-order data tensor $D\in R^{I_{1}\times I_{2}\times \cdots \times I_{K}}$ corrupted by large sparse noise. We assume that the tensor D is intrinsically low rank. Thus, D is decomposed into the sum of a low rank tensor and a noise component, that is,

$$D=M\times_{1}L_{1}\times_{2}L_{2}\cdots \times_{K}L_{K}+E,$$ (40)

where $M\in R^{r_{1}\times r_{2}\times \cdots \times r_{K}}$, $L_{i}\in R^{I_{i}\times r_{i}}$, $E\in R^{I_{1}\times I_{2}\times \cdots \times I_{K}}$, *r*_*i*_ < *I*_*i*_, *i* = 1,2,…, *K*. We can further impose the orthogonal constraints on all mode matrices ***L***_*i*_. To recover the low rank and the sparse terms, we solve the following minimization problem

$$\begin{array}{l} {\text{min}}_{L,M,E}\;{\left\|E\right\|}_{1}\\ \text{s}.\text{t}.\;D=M\times_{1}L_{1}\times_{2}L_{2}\cdots \times_{K}L_{K}+E,\\ \;L_{i}^{T}L_{i}=I_{r_{i}},i=1,2,\ldots ,K, \end{array}$$ (41)

where $L={\left\{L_{i}\right\}}_{i=1}^{K}$. If *K* = 3 and $L_{3}=I_{r_{3}}$, then problem (41) is changed into problem (38).

Without considering the orthogonal constraints in problem (41), we construct its partial augmented Lagrange function:

$$\begin{array}{l} f_{\mu }(L,M,E,Y)\\ ={\left\|E\right\|}_{1}+\frac{\mu }{2}{\left\|D-M\times_{1}L_{1}\times_{2}L_{2}\cdots \times_{K}L_{K}-E\right\|}_{F}^{2}+\langle Y,D-M\times_{1}L_{1}\times_{2}L_{2}\cdots \times_{K}L_{K}-E\rangle . \end{array}$$ (42)

Similarly, we propose an ALM method to solve problem (41). Concretely speaking, if Y is fixed, we alternately update each block of variables by minimizing $f_{\mu }(L,M,E,Y)$ with respect to one argument.

**Computing *L*.** For fixed *j* ∈ {1, 2,…, *K*}, let ***L***_*j*_ be unknown and other blocks of variables be known. Then, the update formulation of ***L***_*j*_ is in the following:

$$\begin{array}{l} L_{j}:={\text{arg min}}_{L_{j}}\;f_{\mu }(L,M,E,Y)\\ ={\text{arg min}}_{L_{j}}{\left\|\left(D-E+Y/\mu \right)-P\times_{j}L_{j}\right\|}_{F}^{2}\\ ={\text{arg min}}_{L_{j}}{\left\|{\left(D-E+Y/\mu \right)}_{\left(j\right)}-L_{j}{(P{}_{\left(j\right)})}^{T}\right\|}_{F}^{2}\;, \end{array}$$ (43)

where $P=M\times_{1}L_{1}\cdots \times_{j-1}L_{j-1}\times_{j+1}L_{j+1}\cdots \times_{K}L_{K}$. In view of the orthogonal property of ***L***_*j*_, we take

$$L_{j}:=QR\left({\left(D-E+Y/\mu \right)}_{\left(j\right)}P_{\left(j\right)}\right).$$ (44)

**Computing M.** Once ***L***_*j*_ is updated, we immediately compute M. The calculation procedure is as follows

$$\begin{array}{l} M:={\text{arg min}}_{M}\;f_{\mu }(L,M,E,Y)\\ ={\text{arg min}}_{M}{\left\|D-M\times_{1}L_{1}\times_{2}L_{2}\cdots \times_{K}L_{K}-E+Y/\mu \right\|}_{F}^{2}\\ ={\text{arg min}}_{M}{\left\|\left(D-E+Y/\mu \right)\times_{1}L_{1}^{T}\times_{2}L_{2}^{T}\cdots \times_{K}L_{K}^{T}-M\right\|}_{F}^{2}\\ =\left(D-E+Y/\mu \right)\times_{1}L_{1}^{T}\times_{2}L_{2}^{T}\cdots \times_{K}L_{K}^{T}. \end{array}$$ (45)

**Computing E.** Let E be unknown and other variables be fixed. We update E according to the following formulation:

$$\begin{array}{l} E:={\text{arg min}}_{E}\;f_{\mu }(L,M,E,Y)\\ ={\text{arg min}}_{E}\;{\left\|E\right\|}_{1}/\mu +\frac{1}{2}{\left\|\left(D-M\times_{1}L_{1}\times_{2}L_{2}\cdots \times_{K}L_{K}+Y/\mu \right)-E\right\|}_{F}^{2}\\ =S_{1/\mu }\left(D-M\times_{1}L_{1}\times_{2}L_{2}\cdots \times_{K}L_{K}+Y/\mu \right), \end{array}$$ (46)

where $S_{1/\mu }\left(\cdot \right)$ is the tensor generalization of absolute value shrinkage operator (19).

**Computing Y**. The update for Y is as follows

$$Y:=Y+\mu \left(D-M\times_{1}L_{1}\times_{2}L_{2}\cdots \times_{K}L_{K}-E\right).$$ (47)

The complete algorithm description of the robust low rank tensor approximations is omitted due to its similarity with RGLRAM.

## Experiments

### A. Ethics Statement

Some face datasets were used in this paper to verify the performance of our method. These face datasets are publicly available for face recognition research, and the consent was not needed. The face images and the experimental results are reported in this paper without any commercial purpose.

### B. Experimental Results

In this section, we carry out experiments on synthetic data and real-world face datasets, and illustrate the feasibility and effectiveness of the proposed method. The experimental results of RGLRAM are compared with previous state-of-the-art methods: PCAL1 [22], RPCA and GLRAM. Both PCAL1 and RPCA are implemented on each training sample ***D***_*i*_ and the inexact ALM algorithm [17] is employed to solve RPCA. For the aforementioned four methods, the tolerance error *ε* is set to be 10^−8^ and the maximum number of iterations is 1000. All experiments are performed using Matlab R2012a on an Intel Core i3-3220 3.30 GHz machine with 3.48 GB RAM.

#### Synthetic data

In this subsection, we synthesize *N* data matrices ${\left\{D_{i}\in R^{m\times n}\right\}}_{i=1}^{N}$ according to the following formulations: ***D***_*i*_ = ***A***_*i*_ + ***E***_*i*_, where ***A***_*i*_ are low rank and ***E***_*i*_ are sparse. Concretely speaking, ***A***_*i*_ are generated randomly as follows:

$$A_{i}=\text{orth}(U)S_{i}{\left(\text{orth}(V)\right)}^{T}\;,$$ (48)

where $U\in R^{m\times r_{1}^{\prime }}$, $V\in R^{n\times r_{2}^{\prime }}$, $S_{i}\in R^{r_{1}^{\prime }\times r_{2}^{\prime }}$ and their entries are independently drawn from the standard normal distribution. As for each ***E***_*i*_, we first define *N* projection operators $P_{\Omega }{}_{{}_{i}}(\cdot ):R^{m\times n}\to R^{m\times n}$

$${\left(P_{\Omega }{}_{{}_{i}}(X)\right)}_{jk}=\left\{ \begin{array}{l} x_{jk},\;(j,k)\in \Omega_{i}\\ 0,\;(j,k)\notin \Omega_{i} \end{array}\right.$$ (49)

for arbitrary $X={\left(x_{jk}\right)}_{m\times n}\in R^{m\times n}$, where Ω_*i*_ are produced by uniformly sampling from {1,2,…, *m*}×{1,2,…, *n*} with probability *p*, *i* = 1,2,…, *N*. Then we generate $E_{i}^{\prime }\in R^{m\times n}$ by a uniform distribution on the interval (−*a*, *a*). Finally, we set $E_{i}=P_{\Omega }{}_{{}_{i}}(E_{i}^{\prime }),i=1,2,\ldots ,N$. The magnitude of noise is measured by the Inverse Signal-to-Noise Ratio (ISNR) defined as

$$ISNR=\sqrt{\sum_{i=1}^{N}{\left\|E_{i}\right\|}_{F}^{2}}/\sqrt{\sum_{i=1}^{N}{\left\|A_{i}\right\|}_{F}^{2}}$$ (50)

Large value of ISNR means large noise and it is probably disadvantageous to recovering the low rank components.

For each ***D***_*i*_, we can obtain its low rank approximation ${\overset{⌢}{A}}_{i}$ and noise component ${\overset{⌢}{E}}_{i}$ by some method discussed in this paper. The Relative Errors (REs) are adopted to evaluate the recovery performance of the low rank and the sparse components respectively and they are defined as follows:

$$RE1=\sqrt{\sum_{i=1}^{N}{\left\|{\overset{⌢}{A}}_{i}-A_{i}\right\|}_{F}^{2}}/\sqrt{\sum_{i=1}^{N}{\left\|A_{i}\right\|}_{F}^{2}},$$ (51)

$$RE2=\sqrt{\sum_{i=1}^{N}{\left\|{\overset{⌢}{E}}_{i}-E_{i}\right\|}_{F}^{2}}/\sqrt{\sum_{i=1}^{N}{\left\|E_{i}\right\|}_{F}^{2}}.$$ (52)

The smaller the relative error is, the better the recovery performance is.

In detailed implementation, the parameters setting is stipulated as follows: *m* = *n* = 100, *N* = 50, *r*_1_ = *r*_2_ = *r*, $r_{1}^{\prime }=r_{2}^{\prime }=r^{\prime }$. We initialize the parameter *r* to be *r*′ and vary it from 10 to 50 with an interval of 10. Three groups of experiments are designed according to the magnitude of noise, that is, we take *a* = 0.5,5 and 15, respectively. In each group of experiments, we consider two different sampling rates for Ω_*i*_: *p =* 0.1 and 0.2. For fixed *r*, *a* and *p*, the experiments are repeated 10 times and the average results are reported. The experimental results are shown in Tables 1, 2 and 3, respectively.

**Table 1:** Experimental comparison of different methods for a = 0.5.

			PCAL1			RPCA			GLRAM			RGLRAM		
r	p	ISNR	Time(s)	RE1	RE2	Time(s)	RE1	RE2	Time(s)	RE1	RE2	Time(s)	RE1	RE2
10	0.1	0.92	1.67	4.56e-1	5.56e-1	39.10	5.09e-5	5.56e-5	0.17	1.07e-1	9.93e-1	20.90	4.94e-9	3.13e-9
20	0.1	0.46	3.49	2.94e-1	7.22e-1	77.56	8.55e-3	1.88e-2	0.15	9.84e-2	9.76e-1	23.41	5.81e-9	8.74e-9
30	0.1	0.30	5.13	2.24e-1	8.26e-1	77.54	9.28e-2	3.05e-1	0.19	9.54e-2	9.50e-1	27.22	7.10e-9	1.54e-8
40	0.1	0.23	7.17	1.83e-1	8.97e-1	77.96	1.98e-1	8.46e-1	0.20	9.42e-2	9.11e-1	29.83	7.07e-9	2.54e-8
50	0.1	0.18	8.92	1.54e-1	9.45e-1	77.94	2.73e-1	1.50e0	0.23	9.32e-2	8.60e-1	30.56	8.46e-9	3.98e-8
10	0.2	1.29	1.70	6.50e-1	5.63e-1	66.14	5.77e-3	4.46e-3	0.17	1.52e-1	9.93e-1	21.43	5.97e-9	5.00e-9
20	0.2	0.97	3.41	4.20e-1	7.28e-1	69.06	1.04e-1	1.60e-1	0.19	1.46e-1	9.85e-1	22.87	5.76e-9	7.24e-9
30	0.2	0.79	5.23	3.20e-1	8.33e-1	69.20	2.19e-1	5.09e-1	0.19	1.42e-1	9.73e-1	24.45	6.02e-9	1.02e-8
40	0.2	0.67	7.03	2.60e-1	9.02e-1	70.48	2.81e-1	8.72e-1	0.20	1.40e-2	9.58e-1	25.82	7.02e-9	1.39e-8
50	0.2	0.59	8.98	2.19e-1	9.48e0	59.83	3.28e-1	1.27e0	0.21	1.38e-2	9.38e-1	27.48	8.00e-9	2.01e-8

**Table 2:** Experimental comparison of different methods for a = 5.

			PCAL1			RPCA			GLRAM			RGLRAM		
r	p	ISNR	Time(s)	RE1	RE2	Time(s)	RE1	RE2	Time(s)	RE1	RE2	Time(s)	RE1	RE2
10	0.1	9.12	1.64	4.72e0	5.80e-1	33.48	1.50e-4	1.65e-5	12.31	1.74	0.99	18.57	5.19e-8	2.00e-9
20	0.1	4.57	3.04	3.11e0	7.62e-1	64.01	1.01e-2	2.21e-3	1.01	1.20	0.97	18.81	2.52e-8	3.52e-9
30	0.1	3.81	4.61	2.36e0	8.66e-1	61.10	1.21e-1	3.97e-2	0.78	1.13	0.96	21.56	2.42e-8	3.53e-9
40	0.1	3.30	6.49	1.89e0	9.30e-1	56.99	2.70e-1	1.83e-1	0.68	1.10	0.94	23.13	2.32e-8	4.44e-9
50	0.1	1.87	8.32	1.58e0	9.68e-1	52.65	3.87e-1	2.12e-1	0.46	0.98	0.85	27.02	2.42e-8	9.23e-9
10	0.2	12.85	1.57	6.56e0	5.69e-1	66.50	6.84e-3	5.31e-4	12.67	2.39	0.99	19.75	3.61e-8	4.78e-9
20	0.2	9.66	3.11	4.33e0	7.49e-1	65.32	1.42e-1	2.19e-2	13.80	2.19	0.98	20.79	3.76e-8	4.27e-9
30	0.2	7.87	4.65	3.30e0	8.56e-1	62.42	3.38e-1	7.85e-2	12.69	2.03	0.97	22.58	3.54e-8	4.63e-9
40	0.2	6.71	6.23	2.66e0	9.23e-1	59.88	4.68e-1	1.45e-1	10.92	1.92	0.95	23.76	3.35e-8	4.95e-9
50	0.2	5.88	8.03	2.21e0	9.63e-1	57.37	5.54e-1	2.15e-1	9.13	1.82	0.93	24.91	3.29e-8	6.06e-9

**Table 3:** Experimental comparison of different methods for a = 15.

			PCAL1			RPCA			GLRAM			RGLRAM		
r	p	ISNR	Time(s)	RE1	RE2	Time(s)	RE1	RE2	Time(s)	RE1	RE2	Time(s)	RE1	RE2
10	0.1	27.32	1.56	1.41e1	5.74e-1	36.24	3.69e-4	1.34e-5	16.35	4.78	0.99	17.46	1.46e-7	2.08e-9
20	0.1	13.74	2.99	9.22e0	7.53e-1	61.86	1.04e-2	7.61e-4	19.56	4.07	0.96	16.24	7.55e-8	3.71e-9
30	0.1	9.13	4.58	7.00e0	8.58e-1	58.91	1.23e-1	1.35e-2	17.35	3.70	0.92	17.04	7.04e-8	4.05e-9
40	0.1	6.85	6.30	5.65e0	9.24e-1	55.63	2.76e-1	4.04e-2	19.65	3.46	0.88	25.55	5.38e-8	5.03e-9
50	0.1	5.48	7.95	4.73e0	9.64e-1	52.59	4.00e-1	7.30e-2	18.70	3.29	0.82	26.83	5.29e-8	7.86e-9
10	0.2	38.56	1.55	1.96e1	5.66e-1	55.08	7.07e-3	1.82e-4	13.33	6.65	0.99	19.00	5.87e-1	1.53e-2
20	0.2	28.98	3.01	1.29e1	7.43e-1	52.76	1.43e-1	7.39e-3	16.17	6.14	0.97	19.48	2.94e-1	7.63e-3
30	0.2	23.62	4.66	9.82e0	8.50e-1	49.90	3.52e-1	2.72e-2	18.88	5.81	0.96	20.72	1.96e-1	5.09e-3
40	0.2	20.14	6.32	7.95e0	9.18e-1	48.19	4.89e-1	5.05e-2	19.58	5.56	0.94	24.19	9.86e-1	9.04e-2
50	0.2	17.66	7.97	6.64e0	9.60e-1	46.52	5.82e-1	7.51e-2	19.80	5.36	0.91	26.38	1.53e+0	1.68e-1

From these three tables, we have the following observations. (I) For given levels of noise, PCAL1 cannot effectively recover the low rank and the sparse components although it has superiority in running time. The smallest relative error of PCAL1 is 0.154 and the values of RE1 is larger than or equal to 1.58 when *a* = 5 or 15. To some extent, the failure of PCAL1 is resulted in the relatively large ISNR. (II) RPCA achieves good recovery performance for small *r* and has strong robustness to large sparse noise. When *r* = 10, the largest relative error of RPCA is 0.00644, which indicates that RPCA obtains satisfactory recovery performance. However, RPCA deteriorates drastically with the increasing of *r*. Besides, it also has the longest running time. (III) For relatively small noise corruptions (i.e. *a* = 0.5), the RE1 values of GLRAM are grudgingly acceptable while the RE2 values are unacceptable. In the presence of medium or large noise corruptions (i.e. *a* = 5 or 15), GLRAM can not effectively recover both the low rank and the sparse matrices. (IV) If *a* ∈ {0.5, 5} or *p* = 0.1, the smallest relative error of RGLRAM is 1.46×10^−7^, which means RGLRAM almost exactly recovers both the low rank and the sparse components. The ISNR corresponding to the case (*p*,*a*) = (0.2,15) is so large that RGLRAM can not effectively the low rank and the sparse terms. With regard to the running time, RGLRAM is generally longer than GLRAM and shorter than RPCA. In summary, RGLRAM is the most effective method for recovering the low rank components among these four methods.

#### Sensitivity to initialization and parameter choice

This subsection will evaluate the sensitivity of the ALM algorithm to the initialization of {***L***, ***R***, ***E***, ***Y***} and the choice of {*r*_1_, *r*_2_}. We still use the artificial datasets generated by the manner of the previous subsection.

A simple initialization strategy for {***L***, ***R***, ***E***, ***Y***} is adopted in Subsection 7.1, that is, both ***L*** and ***R*** are orthogonalized randomly while ***E***_*i*_ and ***Y***_*i*_ are initialized to be zero matrices. For the special synthetic data, we observe from Tables 1, 2 and 3 that RGLRAM successfully recover the low rank and the sparse components if *a* ∈ {0.5, 5} or *p* = 0.1. Under this situation, the standard deviation of running time varies in the interval [0.76, 4.58], that of RE1 varies in the interval [1.77×10^−9^, 7.52×10^−8^] and that of RE2 varies in the interval [9.02×10^−10^, 1.43×10^−8^]. These observations on some datasets illustrate that the ALM algorithm is not very sensitive to the random initialization of ***L*** and ***R***. Subsequently, we will discuss the sensitivity of the ALM algorithm to the initialization of ***E*** and ***Y***.

**Fig 1:**
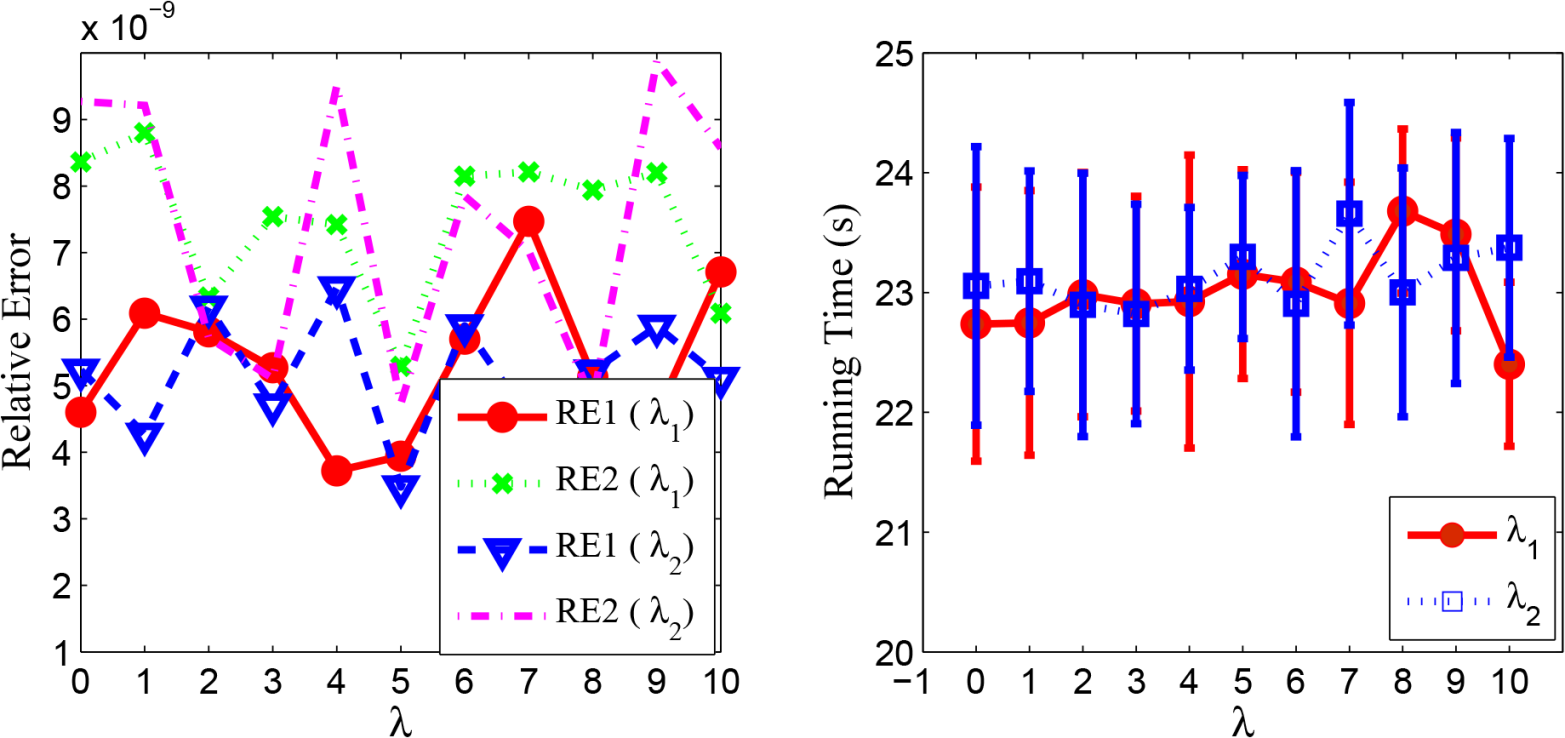
Experimental results of RGLRAM with different initializations on E and Y. The left represents the relative errors vs. *λ* and the right represents the running time vs. *λ*.

In previous experiments, we initialize ***E***_*i*_ and ***Y***_*i*_ with zero matrices. Nowadays, a new initialization method is considered as below: ***E***_*i*_ = *λ*_1_ × *randn*(*m*, *n*), ***Y***_*i*_ = *λ*_2_ × *randn*(*m*, *n*), where *λ*_1_ and *λ*_2_ are two given real numbers, *i* = 1,2,…, *N*. It is obvious that ***E***_*i*_ and ***Y***_*i*_ are zero matrices if *λ*_1_ = *λ*_2_ = 0. We consider 11 different values for *λ*_1_ and *λ*_2_, that is, *λ*_1_, *λ*_2_ ∈ {0,1,…,10}. For the convenience of designing experiments, we only consider two taking value situations of *λ*_1_ and *λ*_2_: varying the value of one parameter while letting the other be zero. The generating manner of ${\left\{D_{i}\right\}}_{i=1}^{N}$ is the same as Subsection 7.1. The other parameters are set as follows: *r*_1_ = *r*_2_ = *r* = 20, *p* = 0.1, *a* = 0.5. The experiments are repeated 10 times for given parameters and the experimental results are described in Fig 1, where the left shows the relative errors under different *λ*, and the right plots the error bar of running time with varying *λ*. From Fig 1, we can see that the relative errors lie in the range from 10^−9^ to 10^−8^, which means RGLRAM obtains the relatively stable values. In addition, RGLRAM has small fluctuation in the average running time and the corresponding standard deviation is no more than 1.22 seconds. The above experiments implies that for our synthetic data, the ALM algorithm is also not too sensitive to the random initialization of both ***E*** and ***Y***.

**Fig 2:**
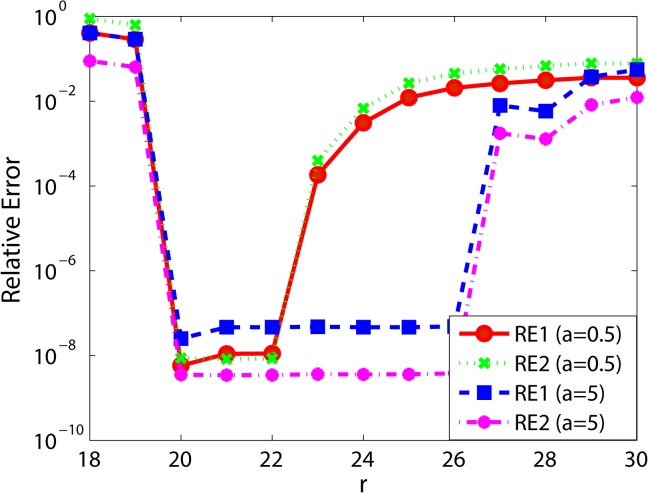
Comparison of relative errors with different r.

Next, we investigate the influence of *r* on the recovery performance. For this purpose, we consider the case that *p* = 0.1, *r*′ = 20 and *a* ∈ {0.5, 5}. We vary the value of *r* from 18 to 30. Fig 2 illustrates the comparison of relative errors with different *r*. We can see from this figure that both RE1 and RE2 of RGLRAM are less than 0.0068 when *a* = 0.5 and 20 ≤ *r* ≤ 24, and less than 0.0080 when *a* = 5 and 20 ≤ *r* ≤ 28. These observations on synthetic data show RGLRAM can successfully recover the low rank and the sparse components when the rank *r* belongs to some interval. Furthermore, *r* should be large than or equal to *r*′ to obtain better recovery performance. It is probable that the parameter *r* has large taking value interval in the presence of large noise. In a word, RGLRAM is not very sensitive to the choice of *r* on the synthetic data generated by our paper.

#### Model evaluation and computation complexity verification

In this subsection, we will evaluate the generalization performance of RGLRAM and verify its computation complexity through extensive experiments.

The *S*-fold cross validation is employed to evaluate the generalization ability of RGLRAM. We first randomly partition the given samples into *S* equal sized subsamples. Then one subsample is chosen as the validation for testing the model, and the remaining *S*-1 subsamples are used as the training set. This validation process is repeated *S* times. Finally, we average the *S* results from the folds. In implementation, we first obtain two orthogonal matrices ***L*** and ***R*** by learning from the training set, and then apply them to the testing set.

We still use the synthetic data in Subsection 7.1 and set *N* = 50, *p* = 0.1, *a* = 5, *m* = *n* = *r*, *S* = 10. For a test sample ***D***_test_ = ***A***_test_ + ***E***_test_, we hope to obtain its low rank approximation ***LM***_test_ ***R*** ^***T***^, where ***A***_test_ and ***E***_test_ are the low rank and the sparse components of ***D***_test_ respectively. The optimal ***M***_test_ can be obtained by solving the minimization problem:

$$\begin{array}{l} {\text{min}}_{M_{\text{test}},E_{\text{test}}}\;{\left\|E_{\text{test}}\right\|}_{1}\\ \text{s}.\text{t}.\;D_{\text{test}}=LM_{\text{test}}R^{T}+E_{\text{test}}. \end{array}$$ (53)

This optimization problem can be regarded as the special case of problem (8) and resolved by simply revising the ALM algorithm. The relative error RE1 is employed to evaluate the model, and the training error and the test error are listed in Table 4. It can be seen from this table that RGRLAM not only has very small training error, but also almost exactly recover the low rank components in the test procedure. Hence, RGRLAM has good generalization ability.

**Table 4:** Relative error of the training and the test sets.

*r*	10	20	30	40	50
RE1 for training	1.58e-7	1.51e-7	9.08e-8	1.14e-7	1.58e-7
RE1 for test	2.01e-7	1.48e-7	1.50e-7	1.58e-7	1.77e-7

**Fig 3:**
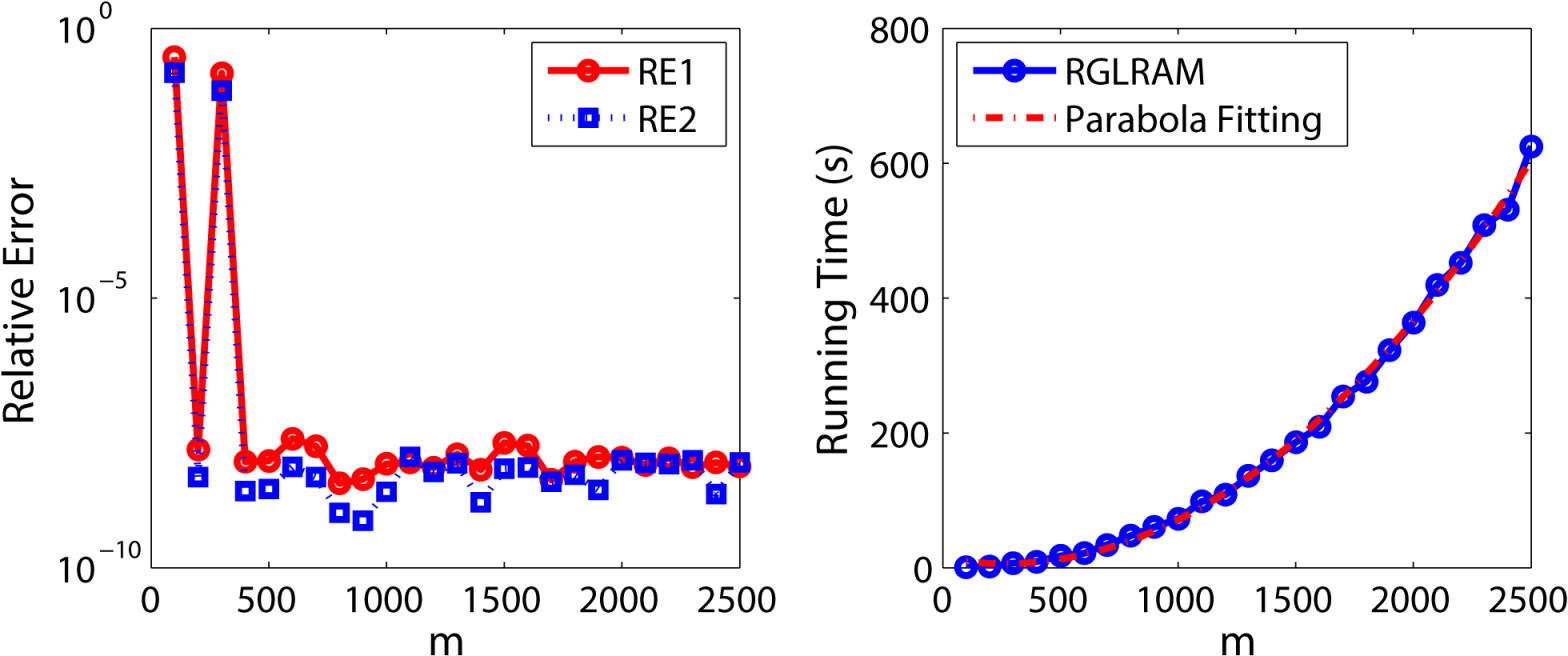
Experimental results of RGLRAM with different m. The left represents the relative errors with different m and the right represents the running time with different m.

Section 4 discusses the computation complexity of the ALM algorithm in one loop. Now, we verify the relationship of running time with the size of matrices and the sample size. To this end, we design two groups of experiments. In the first group of experiments, we study how the total running time changes with the increasing of size of matrices. For the sake of convenience, we let *N* = 1, *a* = 0.5, *p* = 0.1, *m* = *n* = 100*i* and $r_{1}=r_{2}=r_{1}^{\prime }=r_{2}^{\prime }=0.05m$, where *i* = 1,2,…,25. For fixed parameters, the experiments are repeated only once to save time and the experimental results are illustrated in Fig 3, where the right performs a parabola fitting on the discrete running times as a reference curve. We can draw two conclusions from this figure: RGLRAM almost exactly recovers both RE1 and RE2 in most cases; the running time is about a parabola function relation with *m*. The second conclusion is identical with the result of Section 4.

**Fig 4:**
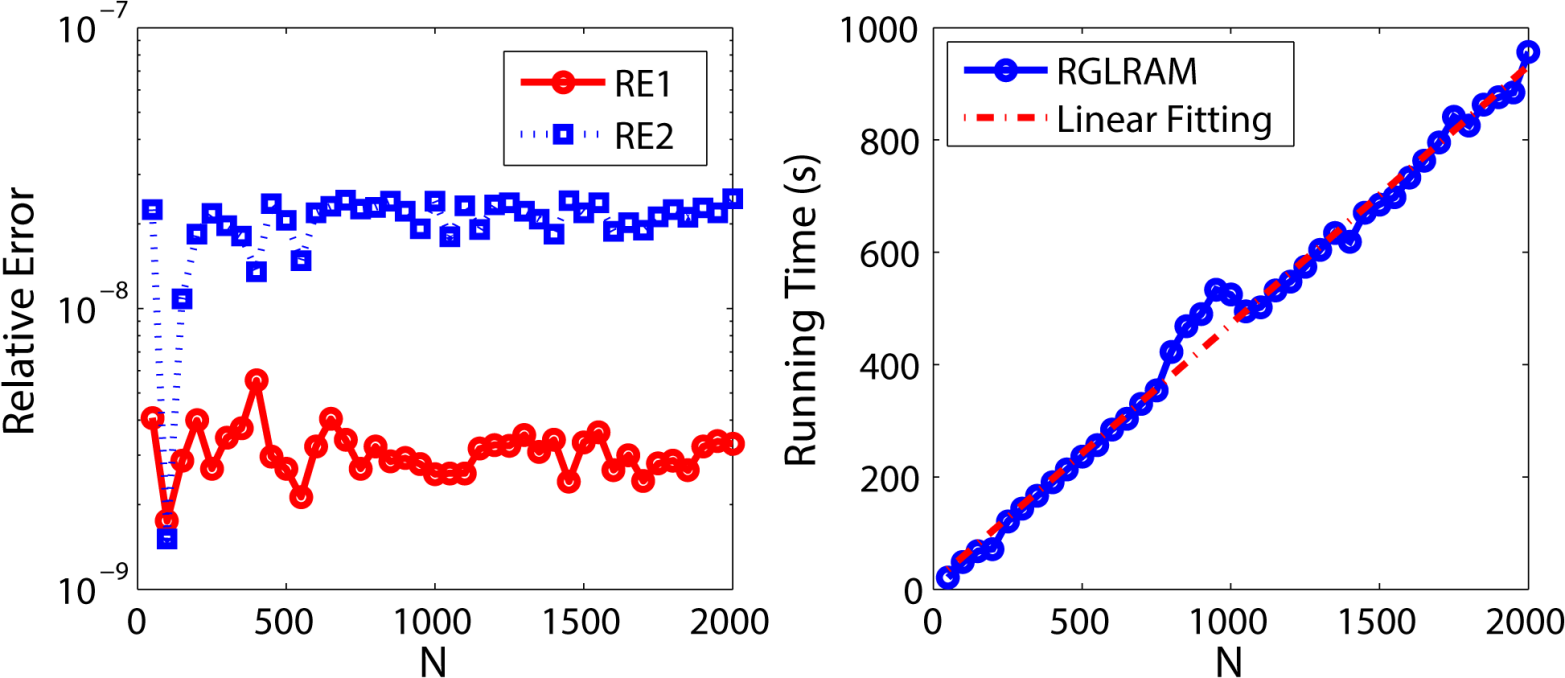
Experimental results of RGLRAM with different N. The left represents the relative errors with different N and the right represents the running time with different N.

The second group of experiments aims to explore the relationship of total running time with *N*. Let $m=n=100,r_{1}=r_{2}=r_{1}^{\prime }=r_{2}^{\prime }=20,a=0.5,p=0.1$ and *N* = 50*i*, where *i* = 1,2,…,40. We conduct the experiments only once for given parameters and outline the results in Fig 4. From the left subplot, we can see both RE1 and RE2 approach to zeros. A linear fitting on the discrete running times is shown in the right subplot. From this plot, we find out that the running time has the linear relationship with *N* on the whole. This conclusion is also in accordance with the result in Section 4.

#### Applications in face images datasets

We carry out the experiments on two well-known face image datasets: Olivetti Research Laboratory (ORL) dataset [24] and Yale face dataset [3]. The former contains the face images of 40 persons, each providing 10 different images. The total 400 images captured at different time have a tolerance for some tilting and rotation of the faces up to 20 degrees. These images show different expressions and facial details, and are normalized to the resolution of 64×64. The latter consists of 165 face images of 15 persons. There are 11 images for each person and they have different facial expression or configuration. Each face image is downsampled into the size of 64×64 for computational convenience.

**Fig 5:**
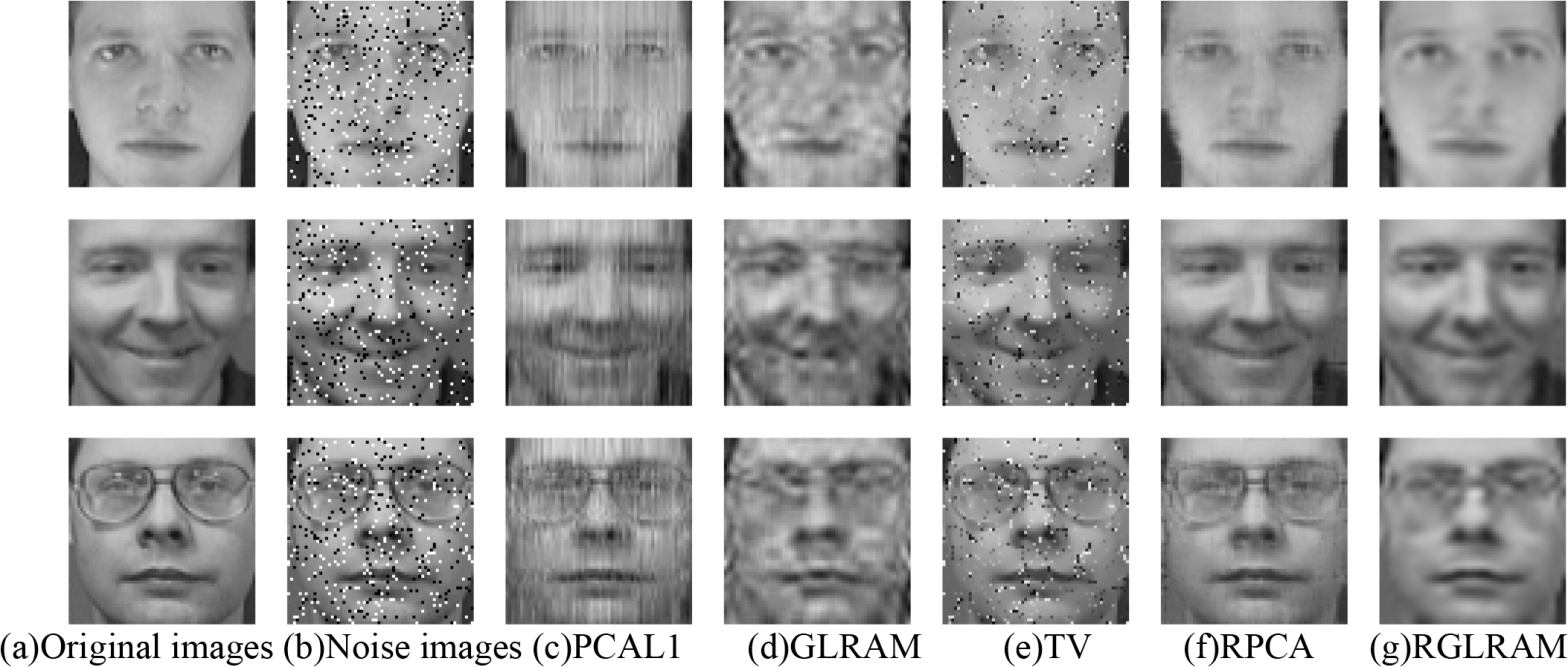
Comparison of low rank recovery and denoising on ORL. This face dataset is open and can be downloaded freely at http://www.cl.cam.ac.uk/research/dtg/attarchive/facedatabase.html. It is publicly available for face recognition research, and the consent was not needed.

**Fig 6:**
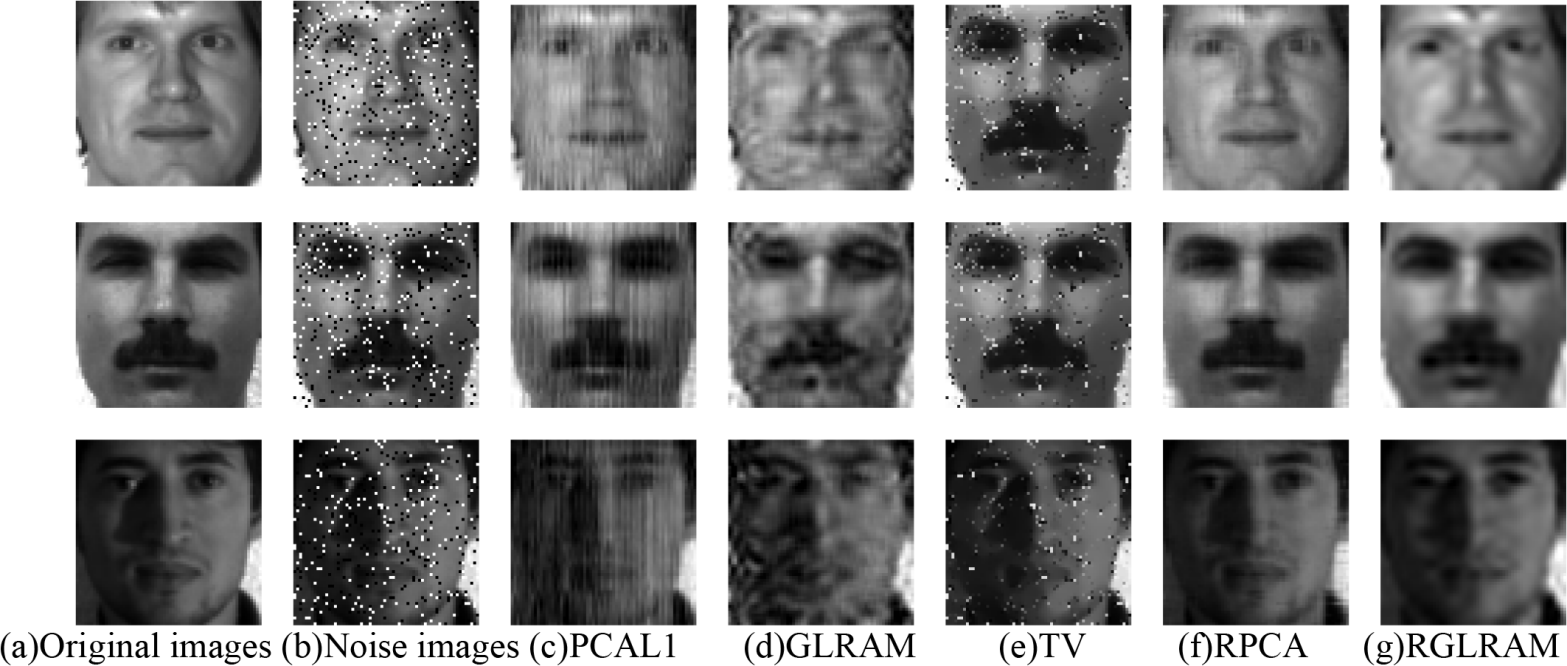
Comparison of low rank recovery and denoising on Yale. **This face dataset is open and can be downloaded freely at** http://vision.ucsd.edu/content/yaleface-database **. It is publicly available for face recognition research, and the consent was not needed.**

We first compare the performance of low rank recovery by adding salt and pepper noise to each image with a density of 0.1. At this moment, the aforementioned two face datasets corrupted by sparse noise are represented by two collections of matrices. We consider four low rank recovery methods and one denoising method listed as follows: PCAL1, GLRAM, RPCA, RGLRAM and Total Variation (TV) [25]. Let *r*_1_ = *r*_2_ = 20 in both GLRAM and RGLRAM, and the low rank to be 20 in PCAL1. Compared with PCAL1, RPCA and TV, both GLRAM and RGLRAM have better compression performance. The compression rate of GLRAM or RGLRAM is 10.08 on ORL, and that of GLRAM or RGLRAM is 9.86 on Yale. The experimental results are partially shown in Figs 5 and 6, respectively.

From the above two figures, we can see that the PCAL1, GLRAM and TV can not efficiently remove the noise and recover facial contour features. In contrast, both RPCA and RGLRAM have better recovery performance. In terms of image reconstruction, RPCA seems to have better quality than RGLRAM, which maybe interpreted as that we choose the relatively small ranks in RGLRAM. Furthermore, RGLRAM has superiority over RPCA in running time and compression ratio. For instance, RGLRAM requires 82.79 seconds on ORL and 32.93 seconds on Yale, and the running time of RPCA is 212.30 seconds on ORL and 86.65 seconds on Yale. To sum it up, RGLRAM has the best performance in recovering low rank components.

Secondly, we compare the values of Peak Signal to Noise Ratio (PSNR) with different rank and noise density, where PSNR is defined as follows:

$$PSNR=10\;{\text{log}}_{10}\left(255^{2}mnN/\sum_{i=1}^{N}{\left\|{\overset{⌢}{A}}_{i}-D_{i}\right\|}_{F}^{2}\right),$$ (54)

where ***D***_*i*_ indicate the image matrices corrupted by noise and ${\overset{⌢}{A}}_{i}$ are the recovered matrices. We set *r*_1_ = *r*_2_ = *r* and vary *r* from 10 to 30 with an interval of 5. The density of salt and pepper noise is denoted by *p*. The experiments are repeated 10 times for fixed parameters and the average values of PSNR are recorded. The PSNR comparison among PCAL1, GLRAM and RGLRAM is shown in Table 5, and the comparison between TV and RPCA is shown in Table 6.

**Table 5:** PSNR comparison among PCAL1, GLRAM and RGLRAM on ORL and Yale.

		ORL			Yale		
r	p	PCAL1	GLRAM	RGLRAM	PCAL1	GLRAM	RGLRAM
10	0.1	15.57	23.56	24.13	14.98	21.65	22.05
15	0.1	15.58	24.22	26.46	14.97	22.82	24.92
20	0.1	15.57	23.72	28.55	14.99	22.52	27.27
25	0.1	15.57	22.61	30.18	14.98	21.49	29.13
30	0.1	15.56	21.38	27.78	14.98	20.35	29.68
10	0.2	12.56	22.34	24.07	11.98	20.19	22.00
15	0.2	12.56	22.07	26.35	11.98	20.35	24.76
20	0.2	12.56	20.88	28.21	11.97	19.38	26.92
25	0.2	12.56	19.50	26.45	11.97	18.20	27.93
30	0.2	12.56	18.25	21.86	11.97	17.10	22.17

**Table 6:** PSNR comparison between TV and RPCA on ORL and Yale.

Method	TV(p = 0.1)	TV(p = 0.2)	RPCA(p = 0.1)	RPCA(p = 0.2)
ORL	12.32	8.31	15.85	13.08
Yale	11.13	7.85	15.10	12.41

By combining Tables 5 with 6, we can see that RGLRAM achieves the best PSNR in all cases. These five methods are sorted in order of decreasing of PSNR as follows: RGLRAM, GLRAM, RPCA, PCAL1 and TV. On the average, the PSNR of RGLRAM is 4.55 large than that of GLRAM on ORL, and 5.28 on Yale. These results show that RGLRAM has the best recovery performance.

## Conclusions

In this paper, we study the model of robust GLRAM and present an iterative algorithm based on the technique of ALM. The proposed method can also be employed to solve the problem of robust SVD or *l*_1_-norm PCA. Then we consider the tensor version of robust GLRAM and propose an iterative scheme to robust tensor approximations. It is illustrated experimentally that our method can recover perfectly both the low rank and the sparse components under some conditions.

Our study so far is limited to recovering the low rank and the sparse components for the collections of low rank matrices with a fraction of their entries being arbitrarily corrupted. It would be interesting to investigate the situation that the noise is the superposition of dense small perturbation and sparse gross errors. Moreover, it still needs further research on robust tensor approximations and robust GLRAM with missing entries.

## Appendix

### A. Matlab Function for RGLRAM

function [L M R E iter] = RGLRAM (D,r1,r2)

%D: an N-by-1 cell arrary of m-by-n matrices

% r1 and r2 are the rank of L and R respectively.

% L and R are orthogonal transformation matrices

% E is noise, D{i} = L*M{i}*R+E{i}

% = = = = = parameters setting = = = = =

mu = 1e-4;mubar = 1e10;

rho = 1.1;epsilon = 1e-8;

maxiter = 1000;

[m n] = size(D{1});N = length(D);

% = = = = = initialization = = = = =

L = orth(randn(m,r1));R = orth(randn(n,r2))';

for i = 1:N

M{i} = L'*D{i}*R';

E{i} = zeros(m,n);

Y{i} = zeros(m,n);

end

normD = 0;

for i = 1:N, normD = normD+norm(D{i},'fro')^2;end

normD = normD^0.5;

% = = = = = loop = = = = =

iter = 0;flag = 1;

while iter< = maxiter & flag

iter = iter+1;

% update for L

L1 = zeros(m,r1);

for i = 1:N

L1 = L1+(D{i}-E{i}+Y{i}/mu)*R'*M{i}';

end

[L LL] = qr(L1,0);

% update for M

for i = 1:N

M{i} = L'*(D{i}-E{i}+Y{i}/mu)*R';

end

% update for R

R1 = zeros(r2,n);

for i = 1:N

R1 = R1+M{i}'*L'*(D{i}-E{i}+Y{i}/mu);

end

[RT RR] = qr(R1',0);

R = RT';

% update for M

for i = 1:N

M{i} = L'*(D{i}-E{i}+Y{i}/mu)*R';

end

% update for E

for i = 1:N

EE = D{i}+Y{i}/mu-L*M{i}*R;

E{i} = max(EE-1/mu,0);

E{i} = E{i}+min(EE+1/mu,0);

end

% update for Y

normErr = 0;

for i = 1:N

temp = D{i}-L*M{i}*R-E{i};

Y{i} = Y{i}+mu*(temp);

normErr = normErr+norm(temp,'fro')^2;

end

normErr = normErr^0.5;

%update for mu

mu = min(rho*mu,mubar);

% = = = Err = =

if normErr/normD<epsilon

flag = 0;

end

end

### B. Demonstration for RGLRAM

clear

m = 100;n = 100;

N = 50;

a = 0.5; p = 0.1;

r1 = 20;r2 = r1;

% = = = = = Generation of D = = = = =

L0 = orth(randn(m,r1));R0 = [orth(randn(n,r1))]';

ErrE = 0;ErrA = 0;

for i = 1:N

H = rand(m,n)>1-p;

E0{i} = (rand(m,n)*(2*a)-a).*H;

M0{i} = randn(r1,r2);

A0{i} = L0*M0{i}*R0;

D{i} = A0{i}+E0{i};

ErrE = ErrE+norm(E0{i},'fro')^2;

ErrA = ErrA+norm(A0{i},'fro')^2;

end

ISNR = sqrt (ErrE/ ErrA);

% = = = = = call RGLRAM = = = = =

t0 = cputime;

[L M R E iter] = RGLRAM (D,r1,r2);

t = cputime-t0;

% = = = = = computation for RE = = = = =

n1 = 0;n2 = 0;

for i = 1:N

n1 = n1+norm(A0{i}-L*M{i}*R,'fro')^2;

n2 = n2+norm(E{i}-E0{i},'fro')^2;

end

RE = [sqrt(n1/ErrA) sqrt(n2/ErrE)]; 
